# Atypical E2f functions are critical for pancreas polyploidization

**DOI:** 10.1371/journal.pone.0190899

**Published:** 2018-01-12

**Authors:** Ramadhan B. Matondo, Eva Moreno, Mathilda J. M. Toussaint, Peter C. J. Tooten, Saskia C. van Essen, Elsbeth A. van Liere, Sameh A. Youssef, Laura Bongiovanni, Alain de Bruin

**Affiliations:** 1 Department of Pathobiology, Faculty of Veterinary Medicine, Utrecht University, Utrecht, The Netherlands; 2 Department of Paediatrics, Division of Molecular Genetics, University Medical Center Groningen, University of Groningen, Groningen, The Netherlands; Centro Nacional de Investigaciones Oncologicas, SPAIN

## Abstract

The presence of polyploid cells in the endocrine and exocrine pancreas has been reported for four decades. In rodents, pancreatic polyploidization is initiated after weaning and the number of polyploid cells increases with age. Surprisingly the molecular regulators and biological functions of polyploidization in the pancreas are still unknown. We discovered that atypical E2f activity is essential for polyploidization in the pancreas, using an inducible *Cre/LoxP* approach in new-born mice to delete ubiquitously the atypical *E2f* transcription factors, *E2f7* and *E2f8*. In contrast to its critical role in embryonic survival, conditional deletion of both of both atypical E2fs in newborn mice had no impact on postnatal survival and mice lived until old age. However, deficiency of *E2f7* or *E2f8* alone was sufficient to suppress polyploidization in the pancreas and associated with only a minor decrease in blood serum levels of glucose, insulin, amylase and lipase under 4 hours starvation condition compared to wildtype littermates. In mice with fewer pancreatic polyploid cells that were fed *ad libitum*, no major impact on hormones or enzymes levels was observed. In summary, we identified atypical E2fs to be essential for polyploidization in the pancreas and discovered that postnatal induced loss of both atypical E2fs in many organs is compatible with life until old age.

## 1. Introduction

Polyploidy has been described in mammals for well over 100 years [1] and is characterized by the addition of one or more complete sets of chromosomes within a cell. Polyploid cells contain either increased number of nuclei per cell or multiple genome duplications within a single nucleus [2]. This phenomenon occurs in plants, flies, worms, and fungi and is well tolerated [3]. In mammals, polyploid cells have been observed in different tissues including the placenta, bone marrow, heart, liver, and pancreas [2, 4]. Interestingly, in the pancreas both the endocrine and exocrine cells undergo programmed polyploidization when mice are weaned resulting in a heterogeneous population of cells with different ploidy status [4, 5]. The biological significance of polyploid cells in the pancreas is not known. However, the timing of the appearance of polyploid pancreatic cells coincides with the formation of polyploid hepatocytes in the liver [2].

Recent studies demonstrated that *E2f* transcription factors are key regulators of polyploidization in the liver and placenta, however, it is unknown as to whether E2fs control the generation of polyploid cells in other tissues, such as the pancreas. Inactivation of transcriptional activators *E2f1/2/3* in trophoblast cells and hepatocytes enhanced polyploidization, whereas deletion of the transcriptional repressors *E2f7/8* prevented polyploidization [6–8]. These studies demonstrated that the antagonistic functions of *E2f1/2/3* and *E2f7/8* are important in controlling the ploidy status of liver and placental cells.

*In vivo* studies in mice and zebrafish have demonstrated that atypical E2fs are not only required for hepatocyte and trophoblast polyploidization, but that they are also essential for embryonic and placental development [6], angiogenesis[9], motor neuron guidance [10], and tumour suppression [11, 12]. Complete loss of *E2f7/8* in mice during early embryonic development resulted *in utero* death [6, 13]. Although the consequences of the loss of atypical E2fs during embryonic development are well documented [8, 13], the effect of synchronized postnatal deletion of *E2f7/8* in mice is currently not known. Here we use inducible mouse models to evaluate the physiological and pathological significance of postnatal *E2f7/8* loss. Surprisingly, synchronized deficiency of atypical E2fs did not have significant effects on postnatal growth and survival. However, the generation of polyploid cells in the pancreas depended on *E2f7/8*. Intriguingly the inhibition of polyploidization, utilizing an inducible mouse model of postnatal *E2f7/8* deletion, had no major effects on the production and release of pancreatic hormones and enzymes.

## 2. Material and methods

### 2.1 Animals

Experiments were performed in accordance with the Utrecht Veterinary Experiment Commission guidelines on animal use in research (the DEC Utrecht), study approval numbers 2012.III.08.083 & 2013.III.10.073. Postnatal induction of *CreERT2* expression was done as described previously [14] through intra-gastric injection of 50μg of tamoxifen in corn oil (Sigma, T5648-1G) per new born mouse per day for three days. Induction of *CreERT2* expression in adult mice started from postnatal day 56 through intraperitoneal injection of 1mg of tamoxifen in corn oil per mouse per day for five days. Mice were housed under controlled experimental conditions of 12hrs light/dark cycle and 21± 1°C, and fed normal laboratory chow (CRM, Tecnilab-BMI) from weaning to old age. Food was either removed early in the morning for four hours (short starvation) or overnight (12 hours starvation) and then animals were sacrificed using CO_2_ method. Some animals subjected to 12-hour starvation were fed with laboratory chow for two hours before were sacrificed. The generation of *E2f7* and *E2f8* knockout mice (conditional and conventional) has been described previously [13]. *CreERT2 [15] and R26R-LacZ*^*LoxP/LoxP*^ mice [16, 17] were derived from Jackson laboratory. Genotyping of mice was performed as described previously [8].

### 2.2 Western blot, immunostaining and β-galactosidase

Western blotting, immunostainings and β-galactosidase were performed as previously described[8]. Antibodies for indicated antigens include *beta-catenin* 1:200 in PBS (AB6302, Abcam), insulin 1:800 in PBS (sc-9168, Santa cruz), glucagon 1:1200 in PBS (PA039-5P, BioGenex), e-cadherin 1:500 in MOM diluent (610182, BD Bioscience), beta-galactosidase 1:1000 in PBS (8559762, MP Biomedical), and amylase 1:5000 in Tris Buffered saline (Sc-46657, Santa Cruz). Reagents: DAPI (5mg/mL) 1:4000 in PBS (D1306, Thermofisher) and 5-Bromo-4-chloro-3-indolyl β-D-galactopyranoside 1mg/mL in PBS (Sigma-Aldrich, B4252-100MG) and mouse on mouse (MOM) basic kit (BMK-2202, Vector Laboratories) according to manufacturer’s descriptions, ABC basic kit 1:25 (PK-4000, Vector Laboratories) and 3–3’-Diaminobenzene (DAB) (SK-4100, Vector Laboratories) in the presence of 0.0005% (v/v) hydrogen peroxide (H_2_O_2_).

### 2.3 Histological image acquisition and processing

Digital bright field images were acquired using Olympus BX46 microscope equipped with DP26 camera and Labsens standard software (Olympus). Immunofluorescence images were acquired using Leica SP II confocal microscope and Leica Application Suite (Leica Microsystems B.V.). Image cytometry analysis of DAPI fluorescence intensity was done by using cell profiler software available at http://cellprofiler.org/ and previously described [18]. Quantification of binucleation using immunofluorescent and e-cadherin stained images obtained from three animals per genotype was done as previously described [8]. Total numbers of binucleated exocrine cells were counted under 63x objective and 1.5 zoom factor using immunofluorescent stained images. Percentage of binucleation was calculated by dividing total binucleated cells to the total number of cells counted in the field multiplied by 100%. Data are presented as average of five counted fields per genotype. Percentage of acinar cells with evidence of mitosis was obtained by using the formula: Total number of mitotic cells in 4–5 randomly selected high power fields (400x maginification) divided by 1000 multiplied by 100%. Other cell types such as duct cells, connective tissues and endothelial cells were excluded in the analysis.

### 2.4 Biochemical analysis

Biochemical analysis for serum glucose, alpha amylase and lipase were performed at Utrecht University Veterinary Diagnostic Laboratory using the automated bio-analyser (AU680, Beckman Coulter) according to manufacturer’s instructions. Serum glucose values were validated using Mouse glucose kit (Cat 81692) kindly provided as gift by Crystal Chem. Insulin was measured using Ultra-Sensitive Mouse Insulin ELISA Kit, (Crystal chem, cat 90080).

### 2.5 Flow cytometry analysis

Prior to FACS analysis, tissues were lysed sequentially in ice cold PBS starting with 1mL pipette tip, followed by 18G and 21G needles respectively attached to a 2mL syringe. Then, nuclei suspensions were mixed with ice-cold absolute ethanol to 70% final concentration and refrigerated for storage. Samples were washed in cold phosphate buffered saline (PBS) by brief centrifugation for five minutes followed by digestion of membranes and cytosolic proteins using 0.5mg/mL pepsin (P700-25g, Sigma) in 0.1N HCL for 20 minutes at room temperature. After brief centrifugation to remove enzymes, pellets were washed with PBS buffer containing 0.1% bovine serum albumin (BSA) and 0.5% Tween once and digestion was completed by re-suspending the pellet in 2N HCL at 37°C for 12 minutes. Samples were equilibrated with borate buffer pH 8.5, and then centrifuged and washed with BSA. Membrane-free nuclei pellets were stained with 5μg/mL propidium iodide (P4170, Sigma) containing 250μg/mL of RNAse (10109169001, Sigma), filtered with 40μm cell strainer (BD 352340, BD Bioscience) and refrigerated overnight. Nuclei were analysed with FACS Calibur and CellQuest software (BD Bioscience) or Flow Jo (FlowJo LLC).

### 2.6 RNA isolation, cDNA synthesis and qPCR

Isolation of RNA, preparation of cDNA and quantitativePCR (qPCR) were performed as described previously [8]. PCR was performed on a BioRad CFX using SYBRgreen Supermix (BioRad). Reactions were performed in duplicate and relative amounts of cDNA were normalized to GAPDH and Actin using the ΔΔCt method.

### 2.7 Statistics

All statistical tests for bar graphs were computed using the Mann-Whitney U test method. Mean and standard deviation were calculated using Microsoft excel (2010) ®.

## 3. Results

### 3.1 Synergistic loss of *E2f*7/8 in multiple organs has no impact on postnatal growth and survival of mice

**Fig 1:**
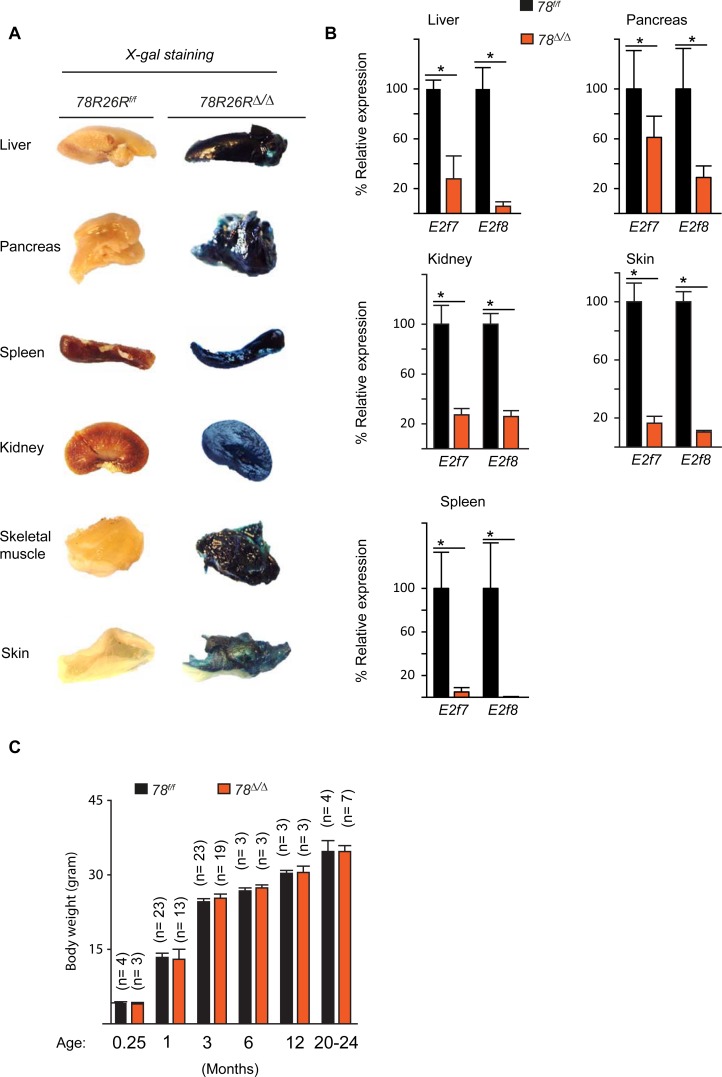
Deletion of atypical E2fs in mice has no effect on growth and survival of mice. (A) Xgal staining of different organs derived from 9 weeks old *CreERT2*^*-/-*^ *E2f7*^*LoxP/LoxP*^ *E2f8*^*LoxP/LoxP*^ *R26R-LacZ*^*LoxP/LoxP*^ mice *(78R26R*^*f/f*^*) and CreERT2*^*+/-*^ *E2f7*^*LoxP/LoxP*^ *E2f8*^*LoxP/LoxP*^ *R26R-LacZ*^*LoxP/LoxP*^ (*78R26R*^*Δ/Δ*^). Newborn pups of both groups were injected with tamoxifen for three days from postnatal day 2. (B) qPCR for *E2f7* and *E2f8* mRNA levels in indicated organs of *78*^*f/f*^ and *78*^*∆/∆*^ mice aged 9 weeks. (C) Body weight measurements over time of *78*^*f/f*^ mice and *78*^*∆/∆*^ mice. Bar graphs represents mean and standard error.

Previous studies have demonstrated that synergistic functions of *E2f7/8* are essential for embryonic and placental development in mice [6, 13]. To investigate whether atypical E2fs are required for postnatal development, we used homologous recombination techniques and *Cre/LoxP* technology to ablate *E2f7/8* in newborn mice. Mice transgenic for the tamoxifen-inducible *CreERT2* at the *Rosa26* locus [15] were crossed to *E2f7*^*LoxP/LoxP*^ *E2f8*^*LoxP/LoxP*^ mice that harbour *LoxP* sites flanking sequences that are required for DNA binding [13]. We confirmed ubiquitous Cre activity by interbreeding the *CreERT2*^*+/-*^ *E2f7*^*LoxP/LoxP*^ *E2f8*^*LoxP/LoxP*^ mice with the Cre reporter mice (*R26R-LacZ*^*LoxP/LoxP*^) in which *LoxP* sites flanking a stop cassette were placed upstream on the *LacZ* gene [16, 17]. Cre expression was induced by intra-gastric injection of tamoxifen into newborn pups on three sequential days (P1-3) [14]. LacZ expression was detected by X-gal staining in one-week-old pups (data not shown) and adult tissues of *CreERT2*^*+/-*^ *E2f7*^*LoxP/LoxP*^ *E2f8*^*LoxP/LoxP*^ *R26R-LacZ*^*LoxP/LoxP*^ of 9 weeks old mice (Fig 1A and S1 Fig). Quantitative PCR (qPCR) at 9 and 16 weeks showed a decrease of *E2f7* and *E2f8* mRNA levels in multiple adult tissues of *CreERT2*^*+/-*^ *E2f7*^*LoxP/LoxP*^ *E2f8*^*LoxP/LoxP*^ mice after neonatal injection of tamoxifen (Fig 1B and S2 Fig). In addition, X-gal and beta-galactosidase staining at 22 and 32 weeks showed that X-gal and beta-galactosidase positive cells were still detectable at 22 and 32 weeks respectively (S3A and S3B Fig)

To investigate the impact of *E2f7/8* loss on postnatal development and survival, *CreERT2*^*+/-*^ *E2f7*^*LoxP/LoxP*^ *E2f8*^*LoxP/LoxP*^ newborn pups (referred to as *78*^*∆/∆*^) and *CreERT2*^*-/-*^ *E2f7*^*LoxP/LoxP*^ *E2f8*^*LoxP/LoxP*^ control newborn pups (referred to as *78*^*f/f*^) were both injected with tamoxifen. Body weights were measured at different time-points up to two years of age. *78*^*∆/∆*^ mice survived to old age similar to *78*^*f/f*^ mice and no significant differences in body weights were observed (Fig 1C). Full necropsy at different stages of postnatal development (1w, 9w, 6m, 9m, 20m, 24m) revealed that neither *78*^*∆/∆*^ mice nor *78*^*f/f*^ mice showed obvious changes in gross morphology (S1 Table). Likewise, analysis of animals that died spontaneously did not reveal genotype-specific macroscopic changes. The pathological changes that were observed in both genotype groups were related to normal aging in mice (data not shown). These findings indicate that functions of atypical E2fs might not be important for postnatal growth and survival, which is surprising because these factors are essential for proper embryonic development and survival.

### 3.2 E2F8 is required for exocrine and endocrine cell polyploidization in the pancreas

**Fig 2:**
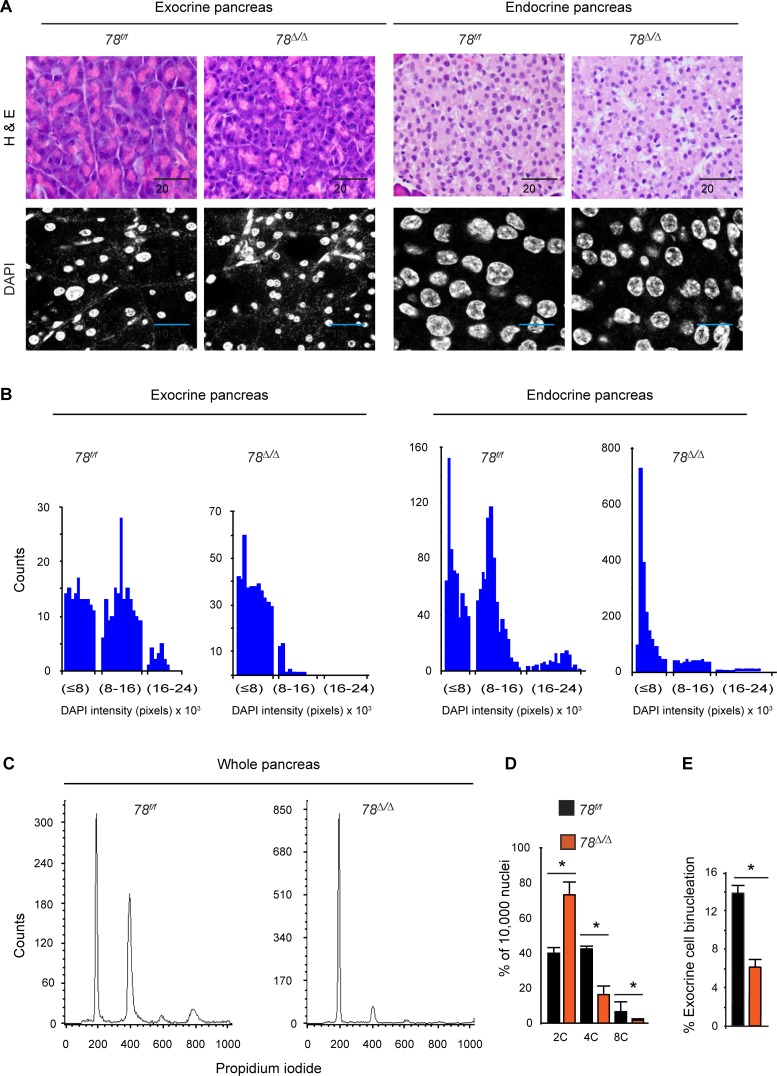
*E2f7/8* are essential for polyploidization in the pancreas. (A) Hematoxylin & Eosin staining (400x magnification), top panel and DAPI (630x magnification, 1.5x zoom factor for exocrine, and 630x, 3x zoom factor for endocrine pancreas) lower panel, showing decreased nuclear sizes in *78*^*∆/∆*^ compared to control *78*^*f/f*^ mice injected with tamoxifen at day 2 after birth and analysed at the age of 22 weeks. (B) Quantification of DAPI fluorescence intensity in pixels. (C) Representative flow cytometry histograms of pancreas tissues showing increased number of diploid cells and decreased number of tetraploid cells in *78*^*∆/∆*^ compared to control *78*^*f/f*^ mice. (D) Summary histograms of flow cytometry profiles for each genetic group (n = 5). (E) Quantification of binucleation in exocrine pancreas; *p<0.05, bar graphs represents mean and standard error.

Previous studies demonstrated that E2F8 is essential for hepatocyte polyploidization [8] and analysis of liver sections from our adult *78*^*∆/∆*^ mice confirmed a reduced rate of liver cell polyploidization in comparison to adult *78*^*f/f*^ mice (S3A and S3C Fig). Interestingly, polyploidization is a developmentally programmed process that starts after weaning of mice and occurs not only in the liver but also in the pancreas [4, 5, 8]. We, therefore, performed histological analysis of pancreas sections of *78*^*∆/∆*^ mice of different age groups, and discovered that the nuclei of exocrine and endocrine cells were smaller in size when compared to pancreatic nuclei of control littermates (Fig 2A and data not shown). To quantify the number of nuclei in relation to nuclear size, we stained pancreatic nuclei with 4',6-diamidino-2-phenylindole (DAPI). In exocrine as well as in endocrine regions of the pancreas we observed that the number of larger nuclei was dramatically reduced in *78*^*∆/∆*^ mice (Fig 2A and 2B). Flow cytometry analysis of isolated pancreatic nuclei stained with propidium iodide revealed that the pancreas of *78*^*∆/∆*^ mice were predominantly composed of diploid (2C) and some tetraploid (4C) cells, whereas the pancreas of control *78*^*f/f*^ mice contained large populations of tetraploid (4C) and octaploid (8C) cells (Fig 2C and 2D). In addition, the number of exocrine bi-nucleated ductal cells was decreased in *78*^*∆/∆*^ mice when compared to *78*^*f/f*^ mice (Fig 2E and S4A and S4B Fig). The number of mitotic figures within the pancreas of 1 week old *78*^*∆/∆*^ mice increased slightly compared to wildtype littermates (S5A and S5B Fig), which indicates that the proliferation rate in *78*^*∆/∆*^ pancreatic cells is elevated possibly to compensate for the smaller cell size. In spite of the observed decrease in ploidy of pancreas and liver in *78*^*∆/∆*^ mice, the liver mass and body mass index was not significantly changed compared to control (S4C and S4D Fig)

**Fig 3:**
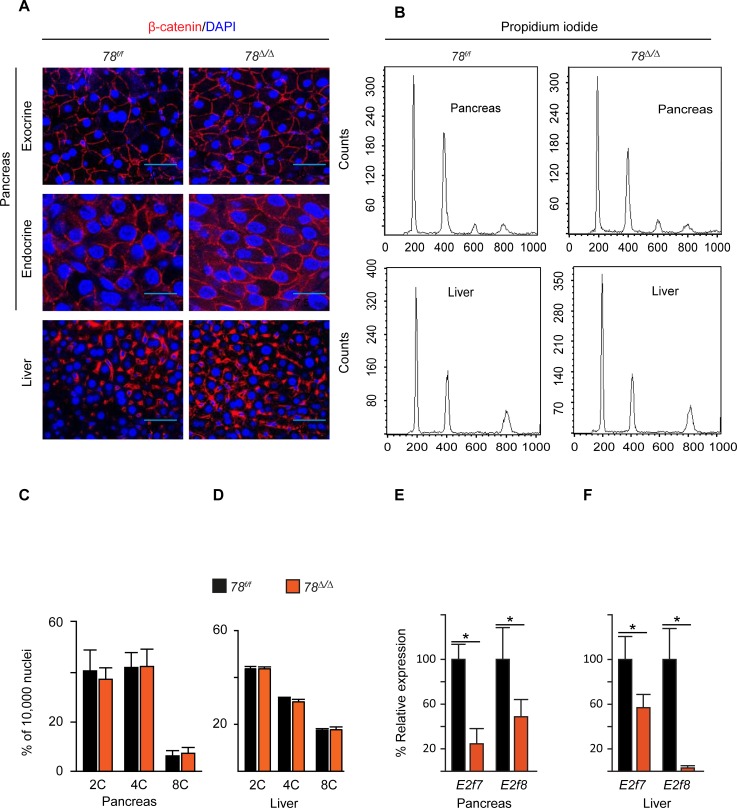
Acute deletion of *E2f7/8* in adult mice has no impact on polyploidy in the pancreas and liver. (A) Beta catenin and DAPI staining of exocrine (630x magnification, 1.5 zoom factor) and endocrine pancreas (630x magnification, 3x zoom factor) and liver (400x magnification) showing similarity in nuclear sizes in the indicated genetic groups. Mice were injected with tamoxifen at the age of 8weeks and analyzed at the age of 22 weeks (B) Representative flow cytometry histograms of pancreas and liver showing similarity in diploid and tetraploid peaks. (C and D) Flow cytometry profiles in pancreas and liver showing similarity in percentage of diploid and tetraploid nuclei (n = 5). (E) qPCR for *E2f7/8* in pancreas (n = 4); (F) qPCR for E2f7/8 in liver (n = 5). Bar graphs represents average and standard error; *p<0.05.

Furthermore, we investigated whether atypical E2fs can also alter the ploidy status of pancreatic cells once polyploid cells have been generated. Since the onset of polyploidization in the pancreas and liver occurs at three weeks of age, we injected tamoxifen into 8-week old mice for five consecutive days to induce deletion of the atypical E2fs. Similar to previous findings in liver [7], post weaning deficiency of *E2f7/8* did not have significant effects on number of diploid, tetraploid and octaploid cells in the pancreas and liver (Fig 3A–3F).

Next, we investigated whether individual deletion of *E2f7* or *E2f8* can alter pancreas polyploidization. Analysis of conventional *E2f7*^*-/-*^ and *E2f8*^-/-^ knockout mice revealed reduced polyploidy in the pancreas of *E2f7*- as well as *E2f8*-deficient mice (S6A–S6F Fig).

Together these findings demonstrate that *E2f7* and *E2f8* are essential for the generation of developmentally-programmed polyploid cells in the endocrine and exocrine pancreas. Interestingly, loss of atypical E2fs did not inhibit polyploidization in megakaryocytes or cardiomyocytes (data not shown), indicating that atypical E2fs act in a tissue-cell type specific manner in the regulation of polyploidy.

### 3.3 Polyploidization has no major impact on pancreatic hormone and enzyme production

**Fig 4:**
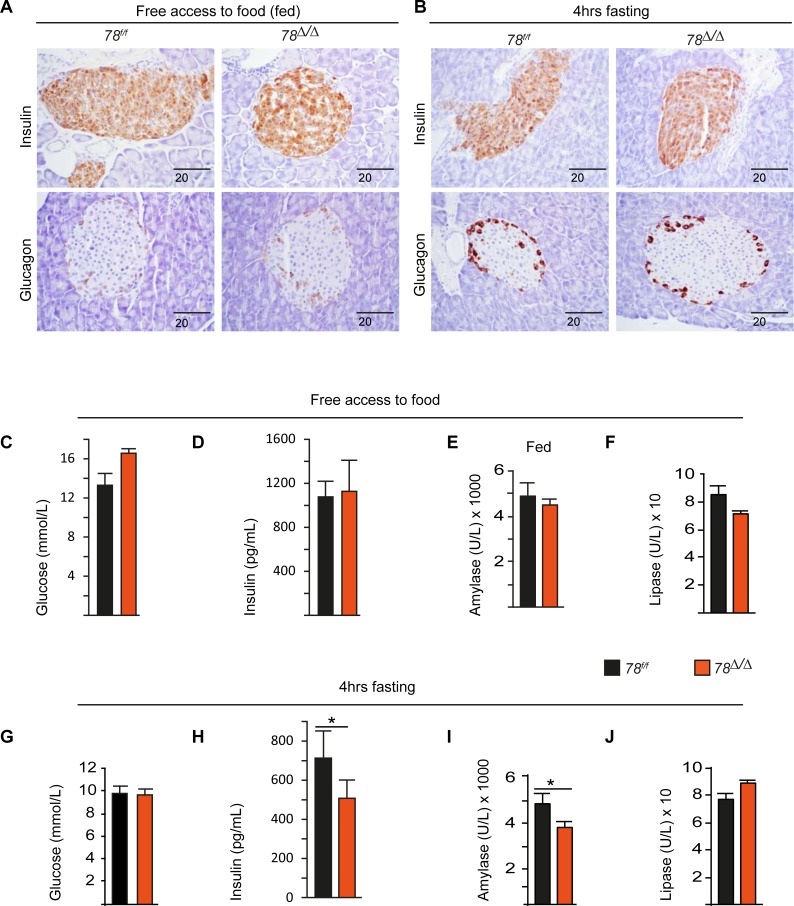
Response to energy stress in *E2f7/8-*deficient pancreas. (A) Immunostaining for insulin and glucagon under fed conditions in mice injected with tamoxifen at the age of 1 week and analysed at the age of 22 weeks. (B) Immunostaining of insulin and glucagon after 4hrs of starvation. (C-F) Serum biochemical parameters under normal feeding conditions. (G-J) Serum biochemical parameters following 4hrs of starvation. Bar graphs represents average and standard error, *p<0.05.

Polyploidization is a common feature under physiological and pathological conditions in multiple mammalian tissues. However, its role in mammalian physiology and pathology is not clearly understood [2, 8]. One of the proposed advantages of polyploidization is to enhance cell function under stress [19, 20]. We therefore evaluated the consequences of reduced ploidy on the function of exocrine and endocrine pancreas cells under normal and stress conditions induced by a short (4hrs) or long (12hrs) period of fasting. We could not detect significant differences in the expression of the endocrine pancreatic hormones glucagon and insulin in α-cells and β-cells respectively when mice were fed *ad libitum* or subjected to 4hr-fasting (Fig 4A and 4B). Furthermore, we measured serum levels of secreted glucose, insulin, amylase and lipase and, in mice fed *ad libitum*, no major differences were observed between the genetic groups (Fig 4C–4F). Following 4hr-fasting, secreted insulin and amylase levels were lower in *78*^*∆/∆*^ mice compared to the control mice, whereas glucose and lipase levels did not differ (Fig 4G–4J).

**Fig 5:**
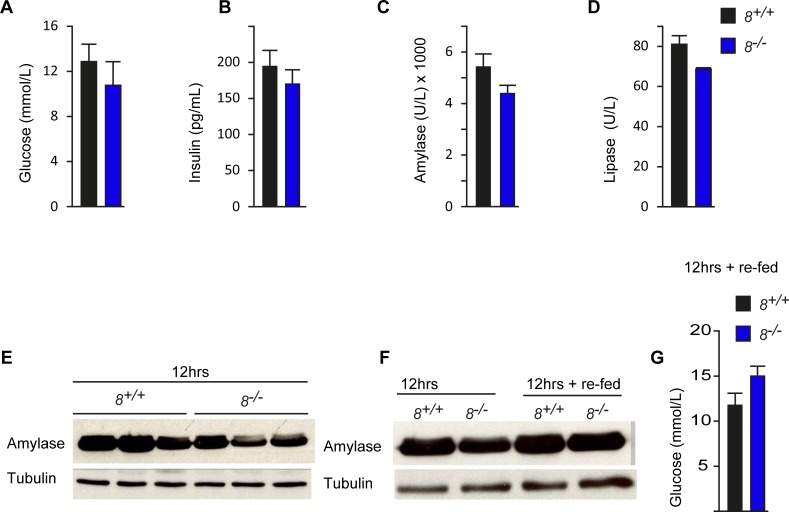
Analysis of glucose, insulin, amylase and lipase in conventional E2f8 knockout mice under starving condition. (A-D) Comparison of serum biochemical parameters after 12hrs of starvation. (E and F) Amylase protein in pancreas of indicated genotypes after 12hrs of starvation or 12hrs starvation. (G) Serum glucose levels after 2hrs of re-feeding. Bar graphs represents average and standard error, *p<0.05.

As *E2f8* deletion alone is sufficient to reduce polyploidization in the pancreas (S6A–S6F Fig), we also measured pancreatic hormone/enzyme production in conventional *E2f8*^*-/-*^ knockout mice. 8 weeks old mice with germline deletion of *E2f8* were fasted for 12hrs and then sacrificed or re-fed for 2hrs before they were sacrificed. Fasting induced a modest but not significant decrease in serum glucose, insulin, amylase and lipase in *E2f8* deficient mice compared to controls (Fig 5A–5D). To confirm whether decreased serum amylase is due to decreased amylase protein expression within the exocrine pancreas, we analysed pancreatic amylase protein levels by western blot from mice which were either starved for 12hrs or starved for 12hrs and then re-fed for 2hrs. Interestingly, when fasted for 12 hours *E2f8*^*-/-*^ mice showed reduced amylase protein levels compared to controls (Fig 5E). The amount of amylase in both genetic groups was similar when mice were re-fed for 2hrs (Fig 5F) although this recovery was accompanied by increased serum glucose levels (Fig 5G) in *E2f8*^*-/-*^ mice compared to controls. In summary, under physiological conditions we could not detect major differences in expression and secretion of pancreatic hormones and enzymes. However, when mice deficient for atypical E2fs were stressed through periods of fasting, amylase and insulin levels were mildly reduced compared to control animals, indicating that polyploidy might contribute to the efficient production of hormones and enzymes in the pancreas under stress conditions. Alternatively, non-polyploid dependent atypical E2f functions could contribute to this phenotype.

## 4. Discussion

In this study we identify *E2f7/8* are required for polyploidization in the pancreas. The *E2f7/8* transcription factors are known to repress the expression of target genes that are important for cell cycle progression. The same set of target genes are also regulated by activator E2fs (*E2f1/2/3*). The appropriate balance between activator E2fs and atypical repressor E2fs is critical for the coordinated oscillation of E2f target gene expression to allow cell cycle progression and cell division [21]. Importantly, balanced E2f activity is not only critical for normal cell cycle progression but is also required for abortive cell cycles, for example during cytokinesis, leading to formation of polyploid cells [7, 8]. Inactivation of atypical repressor E2fs in pancreatic cells, hepatocytes, and trophoblast cells prevents polyploidization and is accompanied by upregulation of cell cycle gene expression. In contrast, loss of activator E2fs results in reduced E2F target expression and enhances polyploidy in the pancreas, liver and placenta [7, 8, 22]. Interestingly, generation of other mammalian polyploid cells such as megakaryocytes and cardiomyocytes occurs independent of atypical E2fs and their regulatory mechanism remains obscure. Wide-spread loss of *E2f7/8* had no major impact on all examined organs (S1 Table) and had no effect on fertility (data not shown).

The common origin of liver and pancreas, that are derived from the ventral foregut endoderm during embryonic development [23], might explain why atypical E2fs are required for polyploidy in both organs. Furthermore, developmentally-programmed polyploidy in liver as well as in pancreas starts after weaning of mice and thereafter each organ shows different distributions of ploidy classes as the animal ages [8, 24]. The appearance of binucleated cells occurs through incomplete cytokinesis and atypical E2fs have been shown to downregulate directly the expression of genes involved in cytokinesis through transcriptional repressor mechanism [8]. Importantly atypical E2F activity is required to induce polyploidy during weaning because induced inactivation of *E2F7/8* in newborn pubs blocks formation of polyploid cells in the pancreas and the liver (Fig 2A–2E and S3A–S3C Fig) [7, 8, 11]. In contrast, inactivation of atypical E2fs in both organs during adulthood has no impact on polyploidy and does not cause a reversal of polyploid cells towards diploid cells (Fig 3A–3F) [7, 11]. Ploidy reversal has been previously described in the liver [25], but its mechanisms are unknown and appear to occur independent of atypical E2fs.

One important question still facing this field is whether polyploidization has a physiological role and if loss of ploidy impacts organ function or alters whole body homeostasis. The pancreas plays an important role in regulating carbohydrate metabolism. The exocrine pancreas produces amylase and lipase enzymes important for digestion and absorption of nutrients, while the endocrine pancreas produces insulin and glucagon hormones which regulate the availability of glucose in peripheral tissues. Somatic polyploidization has been suggested to be important for cellular adaptation to stress and energy depletion [19]. We evaluated whether reduced polyploidization affects the secretory functions of exocrine and endocrine pancreatic cells under normal fed conditions or under short and prolonged starvation. It was expected that endocrine hormones would be reduced in polyploid sufficient and deficient groups during starvation. However we have shown that some endocrine hormones and exocrine enzymes are mildly reduced in polyploid deficient compared to control mice (Fig 4C and Fig 5A–5E). This reduction was more pronounced in mice with inducible post-natal deficiency for both *E2f7/8* compared to mice with conventional single deletion of *E2f8*. (Fig 4C and Fig 5A–5E). Reduction in secreted amylase was confirmed to be related to decreased pancreas amylase (Fig 5C–5E). Salivary amylase levels did not differ between control and *E2f7/8* deficient mice (data not shown). The lack of differences in glucose level after starvation might indicate that these mice retained the glucagon-mediated mobilization of stored glycogen (glycogenolysis) and lipids (for gluconeogenesis) as a mechanism for maintaining blood glucose levels. The ability of *E2f7/8* deficient cells to restore decreased amylase protein after re-feeding indicates that these cells retain the ability to adapt to changes in the nutritional status of the body. Overall the inhibition of polyploidy in the pancreas of *E2f7/8* deficient mice has no major impact on the production or secretion of endocrine hormone and exocrine enzymes. The mild changes we observed might be related to the impact of *E2F7/8* activity on pancreas homeostasis itself independent of polyploidy status of the pancreatic cells. Previous studies demonstrated that mice with loss of both *E2f1/2* developed marked enhanced pancreatic polyploidization, diabetes and exocrine pancreas insufficiency [26]. The increased polyploidization within the *E2f1/2* deficient pancreas could be partially rescued by concomitant deletion of *p53*, suggesting that *p53* is also essential for pancreatic polyploidization. These studies suggests E2fs and p53 activity might be required to maintain tissue homeostasis and regulate the synthesis and secretion of pancreatic products [22, 27].

Conventional knockout mice with complete (*E2f7*^*-/-*^*E2f8*^*-/-*^) or partial germline deletion (*E2f7*^*-/-*^*E2f8*^*+/-*^ *or E2f7*^*+/-*^*E2f8*^*-/-*^) display embryonic death, early postnatal mortality, and short life span [13]. In this study we show that induced conditional loss of *E2f7/8* in different organs has no impact on normal postnatal development and survival. Combined ablation of *E2F7/8* after birth does not affect postnatal development and survival. Combined ablation of E2f7/8 after birth does not affect postnatal development and survival or result in organ dysfunction. As expected based on the results of previous studies demonstrating that keratinocyte and hepatocyte-specific loss of *E2f7/8* did not affect organ function, inactivation in liver and skin (leading to a more than 90% reduction in *E2f7* and *8* transcript levels) did not result in organ failure [8, 12]. The lack of major pathological changes in the mice used in this study suggests that the shorter life span observed previously in mice born with partial germ line deletion (*E2f7*^*-/-*^*E2f8*^*+/-*^ *or E2f7*^*+/-*^*E2f8*^*-/-*^) is likely due to congenital developmental defects rather than postnatal defects acquired as a result of *E2f7* and *8* deficiency. However, as conditional gene disruptions are not 100% efficient and the efficiency can vary depending on dose and duration of Tamoxifen [28] we cannot rule out that non deleted cells might contribute to the growth and regeneration of tissues. Nevertheless we demonstrate by utilizing the Cre/LoxP reporter mice that Cre mediated deletion of LoxP site occurred in the majority of cells within the 20 examined organs as illustrated in Fig 1A and S1 Fig. These analyses were performed on mice tissues from mice injected with tamoxifen in the first week and analysed at the age of 9 weeks. In addition, we also analysed the deletion efficiency in the pancreas of 32 weeks old mice by beta-galactosidase immunohistochemistry (S3B Fig). Here we also observed an efficient deletion in the majority of pancreatic cells in these aged mice. This analysis revealed that non deleted cells represent a minority within the examined organs.

## Supporting information

S1 Fig Ubiquitous Cre activity in multiple organs. Xgal staining of additional organs derived from *CreERT2*^*-/-*^ *E2f7*^*LoxP/LoxP*^ *E2f8*^*LoxP/LoxP*^ *R26R-LacZ*^*LoxP/LoxP*^ *(78R26R*^*f/f*^*) and CreERT2*^*+/-*^ *E2f7*^*LoxP/LoxP*^ *E2f8*^*LoxP/LoxP*^ *R26R-LacZ*^*LoxP/LoxP*^ (*78R26R*^*Δ/Δ*^) mice which were injected with tamoxifen at day 2 and analysed at the age of 9 weeks.
(EPS)

S2 Fig RNA expression of E2f7/8 in mouse organs. RNA expression levels of E2f7/8 in mouse tissues from 16 weeks old mice which were injected with tamoxifen at day 2 and analysed at 16 weeks. Bar graphs represents average and standard error.
(EPS)

S3 Fig Beta-galactosidase and flow cytometry analysis. **(A)** Hematoxylin and Eosin staining of liver tissue, smaller nuclei in *78R26R*^*Δ/Δ*^ compared to control (*78R26R*^*f/f*^), top panel; X-gal staining of control *78R26R*^*f/f*^ and (*78R26R*^*Δ/Δ*^), lower panel obtained from 22 weeks old mice (**B**) Beta galactosidase immunohistochemical staining of control and *78R26R*^*Δ/Δ*^ pancreas obtained from 32 weeks old mice (C) Histograms summarizing flow cytometry analysis of liver tissues of the indicated genotypes obtained from 22 weeks old mice; Bar graphs represents average and standard error, *p<0.05.
(EPS)

S4 Fig E2f7/8 deficiency leads to reduced nuclear sizes in pancreas. (A) Immunohistochemistry staining of e-cadherin in exocrine pancreas to mark the membrane in brown colour, counter stained with hematoxylin to indicate cell nucleus in blue colour and (B) immunofluorescence staining for beta-catenin (red) and DAPI (blue). Both staining shows reduced nuclear sizes in *78R26R*^*Δ/Δ*^ mice compared to control. (C) Liver body weight % and (D) body mass index obtained from 22 weeks old mice which were injected with tamoxifen at day 2 after birth.
(EPS)

S5 Fig Increased mitoses in E2f7/8 deficient pancreas. (A) Mitotic figures in insitu X-gal, Hematoxylin and Eosin stained pancreas. Images taken at 100x magnification, inserts at 400x magnification. Arrow head indicate nuclei undergoing mitosis, (B) Bar graph summarizing the mitotic index quantification. Tissues were obtained from one week old mice, Bar graphs represents mean % and standard deviation of mitotic index counted in 4–5 400x microscopic fields.
(EPS)

S6 Fig Reduction in pancreas polyploidization in conventional E2f7 or E2f8 knockout mice. (A-B) and (D-E) are histograms created using CellQuest software (BD Bioscience) showing flow cytometry in conventional E2f7 and E2f8 deleted pancreas at the age of 16 weeks. (C and F) are Bar graphs summarizing flow cytometry data for E2f7 and E2f8 deleted pancreas respectively. All Bar graphs represents mean and standard error, *p<0.05.
(EPS)

S1 Table List of organ(s) analyzed for pathology. (DOCX)

S1 Methods (DOCX)
