# Supplementary material for: Atypical E2f functions are critical for pancreas polyploidization
Source: PLoS One. 2018 Jan 12;13(1):e0190899. doi: 10.1371/journal.pone.0190899 (PMC5766144; doi:10.1371/journal.pone.0190899)
Supplement: S1 Table — (DOCX) [file pone.0190899.s007.docx]

**S1 Table.** List of organ(s) analyzed for pathology

| SN | Organ(s) | SN | Organ(s) |
| --- | --- | --- | --- |
| 1 | Brain & pituitary gland | 14 | Liver & gall bladder |
| 2 | Eyes & Harderian glands | 15 | Pancreas |
| 3 | Zymbal’s gland | 16 | Kidneys & adrenal glands |
| 4 | Nasal turbinates | 17 | Urinary bladder |
| 5 | Tongue & salivary glands | 18 | Testes & epididymis |
| 6 | Lymph nodes | 19 | Prostate & seminal vesicles |
| 7 | Trachea | 20 | Vagina, cervix & uterus |
| 8 | Thymus, thyroid & parathyroid glands | 21 | Fallopian tubes  & ovaries |
| 9 | Lungs &  bronchi | 22 | Skin, mammary & sebaceous glands, white & brown adipose tissues |
| 10 | Aorta & heart | 23 | Sciatic nerves |
| 11 | Spleen | 24 | Skeletal muscles & diaphragm |
| 12 | Esophagus &  stomach | 25 | Bone, bone marrow & sternum |
| 13 | Intestines (duodenum, jejunum,ileum, caecum,  colon, rectum) | 26 | Spinal cord (cervical, mid-thoracic & lumbar region) |
