# Supplementary material for: Atypical E2f functions are critical for pancreas polyploidization
Source: PLoS One. 2018 Jan 12;13(1):e0190899. doi: 10.1371/journal.pone.0190899 (PMC5766144; doi:10.1371/journal.pone.0190899)
Supplement: S1 Methods — (DOCX) [file pone.0190899.s008.docx]

**Methods**

**Animals**

Java applets available at <http://homepage.stat.uiowa.edu/~rlenth/Power/> was used for estimating sample size using student’s t-test method. Number of animals required to achieve a minimum statistical power of 0.8 with 5% error margin was selected.

**Neonatal deletion of *E2f7/8***

Pups were injected with Tamoxifen at postnatal day 2, 3 and 4 after birth, genotyped at day 15 and assigned to groups. Animals were grouped based on genotype (*E2f7/8* deficient as test group and *E2f7/8* competent mice as control group); 24 animals were used per group (*E2f7/8* deficient and control groups). Study was done in two phases, first as a pilot and then long term observation; each phase, involving 48 animals. Additional 20% extra animals were added on the course of experiments as replacement for animals died because of fighting or other reasons.

**Deletion of *E2f7/8* in adult mice**

24 animals were used per group (*E2f7/8* deficient and control groups). Animals were genotyped and assigned to groups before Tamoxifen injection. 24 *CreERT2^-/-^* *E2f7^LoxP/LoxP^ E2f8^LoxP/LoxP^ R26R-LacZ^LoxP/LoxP^*  (control) and 24 *CreERT^+/-^* *E2f7^LoxP/LoxP^ E2f8^LoxP/LoxP^ R26R-LacZ^LoxP/LoxP^* mice were injected with Tamoxifen for control and E2f7/8 deletion respectively.

**Housing**

All animals were housed in groups, except when animals were fighting or had injury, they were housed individually. Severely injured animals were euthanized.

**Tamoxifen injection**

Tamoxifen stock solution was prepared as a mixture of 750 microlitre of corn oil and 250 microlitre of 100% ethanol {v/v) to 10mg/mL final concentration and stored in refrigerator before use. Just before use, stock solution was diluted in corn oil to 1mg/ml final concentration. Pre-loaded syringes were kept at room temperature and protected from light for 1hour before injection. Adult mice were injected with 1mg per mouse, volume: 1ml per mouse per injection, once per day for 5 days through intra-peritoneal route using hypodermic needle (25G x 1/2"). Pups were injected 50µg/pup per day for 3 days through intragastric route. All animals were monitored daily for first 7 days for any post injection-adverse effects.
